# Mercury Accumulation in Commercial Varieties of *Oryza sativa* L. Cultivated in Soils of La Mojana Region, Colombia

**DOI:** 10.3390/toxics9110304

**Published:** 2021-11-12

**Authors:** Germán Enamorado-Montes, Brayan Reino-Causil, Iván Urango-Cardenas, Siday Marrugo-Madrid, José Marrugo-Negrete

**Affiliations:** Departamento de Química, Laboratorio de Toxicología y gestión ambiental, Facultad de Ciencias Básicas, Universidad de Córdoba, Carrera 6 No. 77-305, Montería 230002, Córdoba, Colombia; genamoradomontes@correo.unicordoba.edu.co (G.E.-M.); coldbeatle@hotmail.com (B.R.-C.); ivanurango@correo.unicordoba.edu.co (I.U.-C.); smarrugo@correo.unicordoba.edu.co (S.M.-M.)

**Keywords:** rice grains, mercury, accumulation

## Abstract

The Hg accumulation in different commercial varieties of *Oryza* *sativa* L. was evaluated in the region of La Mojana, Colombia, where rice cultivation has become the staple food of the population living in this area. The varieties studied were Fedearroz-473 (FA473), Fedearroz-2000 (FA2000), and Fedearroz-Mocari (FAM). Soil spiked at different Hg levels was evaluated, (130, 800, and 1500 µg kg^−1^) using a 3^2^ factorial design that consisted of 3 (rice varieties) × 3 (Hg contents). The biomass, 1000-grain weight, and the accumulation of Hg in the roots, grains, and husks were determined. The highest biomass was found in the FA473 (308.76 ± 108.26 g), and the lowest was found in FAM (144.04 ± 26.45 g) in the 1500 µg kg^−1^ Hg soil in both cases. The weight per 1000-grains decreased significantly in the soil containing 800 µg of Hg kg^−1^. Hg accumulation in the organs of the evaluated varieties was higher in the roots, followed by in the husks and grains. The Hg in the rice grains of the evaluated varieties presented levels close to the permissible limit of the Chinese standard (20 μg Hg kg^−1^) in the evaluated soils and were only exceeded by FA473. Although in natural soil concentrations, the non-cancer health risk (HQ) from rice consumption was lower for FA473 and FAM; Hg enrichment in the soil of La Mojana region may endanger the health of future populations due to their high consumption of rice.

## 1. Introduction

Mercury (Hg) is one of the most highly studied pollutants because of its impacts on human health and ecosystems worldwide. Currently, its distribution, dynamics, and geochemistry are subjects of particular interest for the scientific community [1]. In terms of sources of Hg contamination, gold mining is the most significant [2]. Recently, a study on Hg in the trophic chain of Colombia has revealed important findings regarding the use of Hg during gold exploitation in different regions of this country [3], confirming what was previously disclosed by other authors, who reported that Colombia is one of the regions with the highest incidence of this dangerous metal in the world [4]. Therefore, in Colombia, Law 1658 from 2013 and the Minamata Agreement in 2018 were established, which propose the total elimination of the use of Hg in mining activities by 2018 and in various industrial activities by 2023 [5].

The La Mojana region is one of the most ecologically, economically, and socially important areas in Colombia, mainly due to the wetland complex of the Momposina depression. Historically, its ecosystems have suffered environmental contamination by toxic metals associated with surrounding mining activities, especially gold mining, which involves the use of Hg to recover this precious metal [3]. Hg mainly enters different ecosystems by transport through rivers, such as the San Jorge, Cauca, and Magdalena rivers. The first two rivers are particularly known as important sites for gold production [3]. Additionally, several studies have revealed a significant degree of Hg contamination in different environmental matrices along these waterways [6,7,8]. Furthermore, for the water bodies of the La Mojana region, where significant levels of this metal have been detected, the accumulation of Hg in the surrounding plant and animal communities and its potential genotoxic effect on the biota [9] have been noted.

The most dangerous Hg exposure pathway for humans is through the intake of contaminated food, mainly because foods such as rice and fish may contain the most dangerous chemical species of the metal, namely methylmercury [2,10], although in trace quantities. For the inhabitants of the La Mojana region, both foods are consumed in the daily diet. In the first half of 2020, La Mojana region contributed to 8.6% of the total 394,421 ha of rice grown in Colombia, reflecting the importance of this crop for the area and constituting a socioeconomic pillar in all the municipalities that make up La Mojana region, to the point that it can be considered the primary source of income that is used to meet the basic needs of this region [11].

Hg can accumulate in the different tissues of *O. sativa* L., even reaching the grain. Recent research indicates that when Hg is present in the soil, regardless of its level, it can reach the grain in variable quantities depending on different factors, especially environmental and genetic factors [12]; some characteristics of the soil affect Hg mobility, among which are pH, redox potential, organic matter content, and cation exchange capacity, the presence of chemicals such as oxides, Se compounds and sulfates, and the presence of Hg-resistant bacteria and fungus [13,14,15,16,17,18,19]. On the other hand, water management regimes can significantly affect Hg mobility in soils; Wang et al. [19] found that the total Hg bioaccumulation in rice tissues was greatly reduced under aerobic conditions compared to flooded treatments in paddy fields and pot experiments in greenhouses. In countries such as China, where the diet is largely based on high rice consumption, the health risk due to the intake of Hg-contaminated food may be higher than the risk due to fish consumption [20].

In Colombia, research conducted by Argumedo-García et al. [21] showed that rice sold in the municipality of San Marcos (Sucre) had Hg levels in the order of parts per billion. Additionally, another study by Argumedo-García et al. [22] showed how Hg levels were maintained in rice sold and consumed in the municipalities of Ayapel and San Marcos, even after cooking.

Given that a large number of *O. sativa* L. [23] varieties are grown in the La Mojana region, the objectives of this study were (i) to evaluate whether Hg concentrations in the roots, husks, and grains of *O. sativa* L. plants cultivated in La Mojana region soils contaminated with Hg can increase as the Hg content in the soil increases and (ii) to determine whether exposure to these Hg concentrations influences the growth and development of the different commercial varieties grown in these soils. These findings would be used to inform the inhabitants of the most appropriate rice varieties for planting that would not constitute a potential risk to their health.

## 2. Materials and Methods

### 2.1. Experiments in Pots

This study was conducted in a greenhouse located at the Faculty of Agricultural Sciences at the University of Córdoba, Colombia, from October 2014 to February 2015. The average temperature was 27.8 °C, the relative humidity was between 76 and 82%, and the location was at 8°55′, 75°49′ W and at an altitude of 20 m [24].

Surface soil (0–30 cm) from a rice-growing site in the La Mojana region was transported to the laboratory in pre-washed polyethylene fiber sacks.

Once it was in the laboratory, the soil was homogenized and sieved through a 4 mm sieve before being subjected to the different experimental treatments and was characterized according to its contents of organic matter (loss on ignition; [25]), Ca, Mg, and K (extractable with 1 M ammonium acetate pH 7; [26]); sulfur (S) (extractable with 0.008 M monophosphate; [26]); total mercury, which was extracted using the EPA 7473 method [27], and phosphorus (P) (Bray II; [26]); and its pH [28] and texture (Bouyoucos method; [26]). Ten kilograms of soil were added to each pot. The pots had a cylindrical form with a nominal volume of 10 L. In this study, the original Hg soil concentration from the La Mojana region was 130 µg kg^−1^ Hg (S130); this non-spiked soil was considered as a control, and although it was not free of mercury, it is important to mention that this concentration is below the Maximum Allowable Concentrations Ranges in Agricultural Soils (500–5000 µg kg^−1^ Hg) but it is slightly above the Crustal Average and World-soil average (70 µg kg^−1^ Hg) [29]. For the initial mercury concentrations of 800 (S800) and 1500 (S1500) μg kg^−1^, the Hg was added using a solution of Hg(NO_3_)_2_ to the original soil containing 130 µg kg^−1^ Hg (S130). To ensure a homogeneous distribution of mercury in the soil, a saturated paste was prepared with a ratio of soil to distilled water of 1:2; this mixture was prepared in plastic containers, stirred manually with wooden paddles until a homogeneous consistency was obtained, then a calculated volume of a Hg(NO_3_)_2_ solution was added, the stirred again, and the soils were stabilized and dried under ambient conditions for 15 days before determining their Hg concentrations. The experiment was conducted under an aerobic water management regime.

### 2.2. Seed Treatment

The commercial rice varieties Fedearroz 2000 (FA2000), Fedearroz 473 (FA473), and Fedearroz Mocarí (FAM) were purchased from the National Federation of Rice Growers of Colombia (Federación nacional de arroceros de Colombia, Fedearroz, Monteria-Colombia). All seeds were submerged in a container filled with distilled water for two minutes, and the floating seeds were discarded and those remaining at the bottom were planted in plastic containers, for a total of four seeds per pot.

### 2.3. Agronomic Management

A fertilization plan was established using the physicochemical analyses of the soil; DAP (diammonium phosphate) and urea were applied 16 days after the emergence of the rice plants as the first fertilization since the phosphorus fertilizer contributed additional nitrogen to that provided by the urea. On days 45 and 80 after emergence, a second and third application of urea and KCl was made to prevent a possible deficiency in the macroelements that are essential for normal plant development. Weed control was carried out manually in each experimental unit. The crop was irrigated, as the experimental units were isolated in plastic containers, and there were no infiltration losses. Water was supplied twice a day, in the morning and afternoon, so there was no water stress.

### 2.4. Estimation of Aboveground Biomass and Weight per 1000 Grains

The biomass of aboveground tissue was determined on a dry matter basis (g) at the end of the harvest cycle by extracting the plants and drying them in a Binder 5–300 °C oven with an integrated timer for four days at 60 °C. The masses of each sample were measured using an OHAUS Adventurer analytical electronic balance, model AP2140 [30]. The weight per 1000 grains as a yield component of the rice was estimated, for which the grains of each treatment were separated from the spike and were weighed on an analytical balance.

### 2.5. Chemical Analysis and Sampling

The rice plants were divided into roots, stems, leaves, grains, and husks. Each organ was dried, weighed, and macerated before its analysis. The Hg levels were only determined in the grains, husks, and roots of the rice plants. Approximately 15 to 20 mg of each organ was weighed to determine the Hg using a Direct Mercury Analyzer (DMA-80 TRICELL, Milestone Inc., Sorisole, Italy). The samples were burned at 650 °C to release the Hg vapors that were trapped in a gold amalgamator, and these were subsequently desorbed for detection at 253.7 nm by atomic absorption spectroscopy according to the specifications established in the EPA method 7473 [27]. The method was validated by the triplicate analysis of certified reference tomato leaves (CRM 1753a, 34 µg kg^−1^). Recovery ranged from 97 to 102%, with a relative standard deviation of 5.35%. The limit of detection, which was calculated as the target concentration determined from the calibration curve at low concentrations plus three times the standard deviation (*n* = 7), was 0.01 ng of total Hg per sample, and the calibration curves showed R^2^ values greater than 0.9995.

### 2.6. Experimental Design

To compare the commercial rice varieties at different soil Hg doses, a 3^2^ factorial design was used. One factor was the three varieties of rice; FA473, FA2000, and FAM, and the other factors were the Hg concentrations in the soil (130, 800, and 1500 µg kg^−1^ of Hg), for a total of 9 treatments, with 3 replicates and a total of 27 experimental units.

### 2.7. Translocation Factor (TF)

The translocation of Hg in the rice crops was calculated using the translocation factor (TF), which was defined as follows:

(1) $$\mathrm{TF}=\frac{C_{Grain}}{C_{Root}}$$

where $C_{Root}$ and $C_{Grain}$ are the concentrations of Hg (μg/kg) in the roots and the grains of the rice, respectively. A TF higher than 1 indicates that the plant translocates metals effectively from the roots to the aerial parts [31].

### 2.8. Health Risk Due to Rice Intake

To evaluate the potential risk to human health from rice consumption, the methodology proposed by the USEPA [32] was used to determine the health risk due to the noncarcinogenic effects of Hg exposure.

Exposure to dietary Hg through rice intake was defined as the estimated weekly intake (*EWI*, μg Hg/kg body weight/week) as follows:

*EWI* = *C* × *IR*/*BW* (2)

where *C* is the mean Hg concentration in the grain for each treatment (μg Hg/g); *IR* is the weekly rice intake rate (g/week), which, for the La Mojana region, the value reported by Argumedo-García et al. [21] of 1218 g/week was used; and the *BW* is the body weight (kg), which was assumed to be 70 kg for this study.

The non-carcinogenic health risk as hazard quotients (*HQ*) was used to assess the potential health risk from the Hg as follows:

*HQ* = *EWI*/*PTWI* (3)

In which the provisional tolerable weekly intake of Hg (*PTWI*) is 4 μg Hg/kg of body weight/week according to the JECFA [33]. An *HQ* ratio of less than one is assumed to be safe according to the risk of noncarcinogenic effects. If the *HQ* exceeds one, there is a possibility that noncarcinogenic effects will occur [20].

### 2.9. Data Treatment

Values are represented as means plus or minus (±) the standard deviation. A two-way ANOVA with interactions and multiple comparisons for pairwise contrasts with Tukey’s test were used as the statistical analysis for the factorial design for the three commercial varieties, which were performed using different doses of mercury in the soil and using a statistical package in SAS Plus 4.1 software (SAS Institute Inc., Cary, NC, USA). All of the analyses were conducted with an alpha level of 0.05.

## 3. Results and Discussion

### 3.1. Soil Characterization

The soil presented a clay loam texture with a slightly acidic pH of 5.70, the percentage of organic matter was 2.51%, and there were high available contents of Ca (16.2 meq Ca/100g), Mg (10.4 meq Mg/100g), and S (55.4 mg kg^−1^), as established by Muñiz-Torres [34]. The concentrations of the available K (124.8 mg kg^−1^) and available P (18.9 mg kg^−1^) were lower than those necessary for the physiological development of the plant [35]. A clay loam soil has a moderate content of clay, which can adsorb metallic cations because it contains minerals that enable their immobilization [36]. However, the acidic nature of this soil can promote the mobility of metal ions, leading to the increased absorption of mercury by the rice plants [37].

### 3.2. Effect of Hg on the Biomass and Weight per 1000 Grains of O. sativa L.

**Figure 1:**
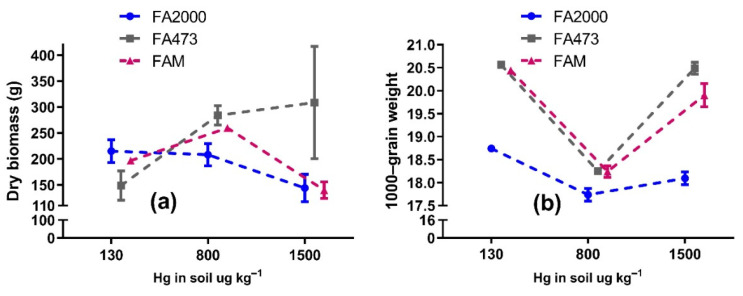
Dry biomass in grams per pot (**a**) and weight (g) per 1000 grains (**b**) of the commercial *Oryza sativa* L. varieties at different Hg levels in La Mojana region soil.

Dry biomass showed differential behavior in response to increasing the soil Hg concentration (Figure 1a), and two-way ANOVA showed that the soil Hg concentration variable and its interaction with the rice variety variable can significantly affect the growth of *O. sativa* L. plants. The biomass of FA473 increased as the soil Hg concentration increased, with the low-level Hg treatment (148.77 ± 28.40 g) being significantly different from the mid- (283.85 ± 18.76 g) and high-level Hg (308.76 ± 108.26 g) treatments. On the contrary, FA2000 showed an inverse trend, decreasing in biomass as the soil Hg concentration increased, presenting 215.00 ± 22.22 g at low-level Hg, 208.24 ± 21.46 g at mid-level Hg, and 144.04 ± 26.45 g at high-level Hg, although these differences in the treatments were not significant. Finally, the highest biomass was recorded for FAM at the intermediate Hg concentration, although the lowest dry biomass of all of the treatments was obtained at the highest level of Hg in the soil, with 140.02 ± 2.36 g.

The results for the FA473 variety were the opposite of those reported by Marrugo-Negrete et al. [30], who found that exposure to different Hg doses (0, 5, 10, 20, 40, and 80 mg.kg^−1^) significantly inhibited the biomass development of *Jatropha curcas*. The FA473 variety exhibited a certain tolerance and favorable adaptation to the Hg since its biomass increased with the concentration of the metal in the soil, which is consistent with the results reported by Sitarska et al. [38] for the species *Lemna minor* and *Salvinia natans.* The authors reported stimulated biomass production due to the presence of mercury. The presence of heavy metals can lead plants to exhibit a tolerance that is sometimes more pronounced as the concentration of the contaminant increases since the presence of heavy metals within the plant cell walls activates the transport of ions to the vacuole thus, avoiding metal toxicity [39,40,41].

The weight per 1000 grains of the three commercial varieties at different initial soil Hg levels was between 17.64 and 20.62 g, with an overall mean of 19.16. (Figure 1b). The variety and soil Hg concentration variables, as well as their interaction, were highly significant (*p* < 0.01) on the weight per 1000 grains of *O. sativa* L. (Table 1). The weight per 1000 grains of the three varieties was significantly lower for the 800 μg kg^−1^ Hg concentration compared to the extreme levels, 130 and 1500 μg kg^−1^ Hg, which presented the highest weights per 1000 grains of the three varieties. According to the results obtained in the present study, it is not clear how the Hg concentration in the soil affects the weight per 1000 grains of *O. sativa* L. Few studies have clarified how abiotic stress (mercury in soil) can affect rice grain yield, and to the best of our knowledge, only Zhu et al. [42] has proposed an explanation arguing that rice grain yield is closely related to a variety of components such as the number of panicles per plant, spikelets per panicle, full spikelets, and 1000 grain weight, which are affected in different ways by the mercury levels in soils.

According to the growth (as dry biomass) and yield component (as 1000-grain weight), the rice varieties FAM and FA473 seem to be better adapted in the soils of the La Mojana region to the mercury levels of this study under controlled conditions. We assume that these rice varieties could have better development in normal field conditions in the soils of the La Mojana region, where Hg concentrations ranging from 5.30 to 383.41 μg kg^−1^ have been reported [43].

### 3.3. Accumulation of Hg in Roots of O. sativa L. Varieties

**Figure 2:**
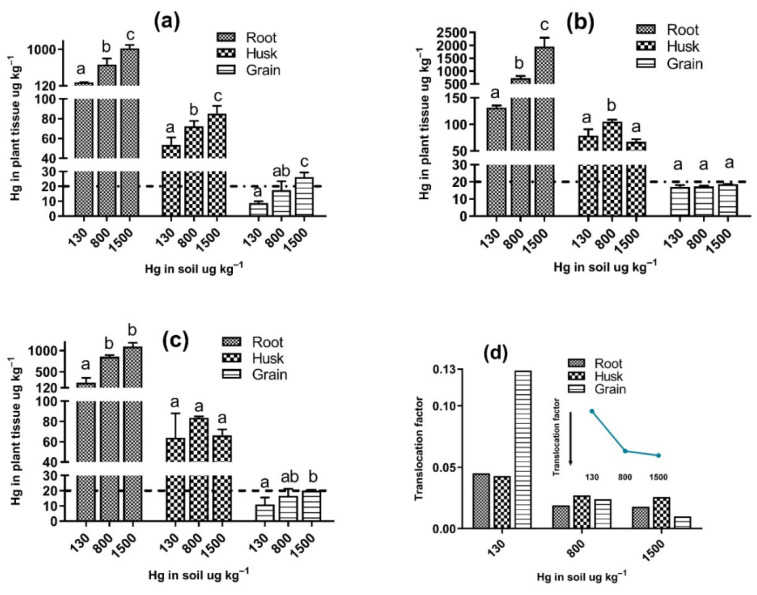
Concentrations of Hg (μg kg^−1^) in the roots, husks, and grains of varieties (**a**) FA473, (**b**) FA2000, (**c**) FAM, and (**d**) *Oryza sativa* L.; translocation factors grown in soils with different Hg levels. Different letters indicate statistically significant differences (α = 0.05). The dashed line at 20 μg kg^−1^ is the threshold established by the Chinese National standard [48].

Figure 2 shows the Hg concentrations in the roots, grains, and husks of the commercial varieties of *O. sativa* L. that were grown in soil contaminated with different mercury levels. In general, the Hg concentrations in the different organs studied here presented the following order: root > husk > grain, with overall means of 739.04, 74.18, and 17.02 µg kg^−1^ Hg, respectively.

The Hg concentration in the *O. sativa* L. roots for the three varieties studied here increased significantly with the Hg concentration in the soil (Figure 2), and only the FAM variety showed no differences between the 800 and 1500 µg kg^−1^ levels according to the a priori comparisons. The highest root concentration (1950.70 ± 347.15 μg kg^−1^) was observed in the FA2000 variety for the 1500 μg kg^−1^ Hg treatment, which was twice as high as that of the other varieties with the same level of Hg in the soil. It is widely accepted that the highest levels of Hg metal are present in the roots [30,44,45,46], mainly because this part of the plant is in contact with the contaminated medium. This distribution could also be a defense mechanism since some authors estimate that approximately 80% of the metal adheres to the cell walls, leading the root to act as a barrier to limit the passage of metal to the aerial parts of the plant [47].

According to the factorial ANOVA, the concentration of Hg in the *O. sativa* L. root is significantly affected by the morphotype and soil Hg concentration variables and the interaction between said variables, resulting in a *p* < 0.005.

### 3.4. Accumulation of Hg in Husk of O. sativa L. Varieties

According to two-way ANOVA, the Hg concentration in the rice husk is significantly influenced by the soil Hg concentration variable and its interaction with the morphotype variable (Table 2). Regarding the Hg accumulation in the *O. sativa* L. husk, each variety presented different behavior. In the FA473 variety, the accumulation of Hg in the husk increased significantly when the Hg in the soil increase (Figure 2a). In contrast, in the FAM variety, increasing the Hg concentration in the soil did not significantly increase the Hg in the husk (*p* > 0.05 in the contrasted pairs) (Figure 2c). The FA2000 variety presented the highest mean concentrations for the husk compared to the other varieties, with the highest being at the intermediate level, with values of 105.32 ± 3.51 μg kg^−1^. We cannot explain the higher Hg accumulation in the husk of the FA2000 variety; however, some authors have proposed that one of the sources of mercury in this organ, in addition to the uptake of Hg from the soil and water by roots, could be attributed to the volatilization of the Hg from soil [49], which is probably due to Hg-resistant bacteria [13]. Even though the dense biomass developed in these treatments can inhibit Hg phytovolatilization, the Hg vapor emissions from the soil in the experiment could be considerable [50,51,52,53].

### 3.5. Accumulation of Hg in Grain of O. sativa L. Varieties

In contrast with Hg in the root, the Hg concentration in the grain was significantly influenced by the morphotype and its interaction with the soil Hg concentration (Table 2).

Although an increase in the Hg concentration in the soil led to a higher accumulation of the metal in the rice grains, these differences were not statistically significant for the FA2000 variety (Figure 2b). While this variety recorded the highest Hg accumulations in the other organs studied here, the mean concentrations of Hg were below 20 μg kg^−1^, the threshold established by the Chinese National standard [48], with this being the only rice variety for which this threshold was not exceeded at any of the soil Hg levels. In the FA473 rice variety, the highest and lowest mean Hg concentrations in the grains from this study were presented, with 8.64 ± 1.28 μg kg^−1^ (only this treatment complies with the maximum level of total Hg in rice prescribed by the European Commission of 10 μg kg^−1^) [54] and 26.15 ± 3.23 μg kg^−1^ for the 130 μg kg^−1^ and 1500 μg kg^−1^ Hg soils, respectively (Figure 2a), with the high-level Hg treatment being significantly different from the mid-and low-level treatments according to the a priori comparisons (*p* < 0.05). In the FAM variety, which was cultivated at 1500 μg kg^−1^ soil Hg, a mean of 20.09 ± 0.48 μg kg^−1^ Hg was accumulated in the grain. However, for the lowest soil Hg concentration treatment, the accumulation of Hg in the grain was below the Chinese threshold.

All of the treatments in the present study presented Hg concentrations in the rice grain that were below those reported by Zhu et al. [42], who found Hg levels in the grain between 52.0 and 623.2 µg kg^−1^ in an experiment developed in China with 38 rice varieties grown in a soil with 4. 72 mg kg^−1^ of Hg; however, in that same experiment, the 38 rice varieties grown in soils with 0.47 mg kg^−1^ of Hg accumulated between 1.1 and 59.0 µg kg^−1^ of Hg in the grain (mean of all treatments of 19.3 µg kg^−1^ of Hg), which are values that are within the range of those observed in the present study. Peng et al. [55] also reported Hg accumulation in rice grain in a similar range to that found in the present study, with 8.5 to 43.2 µg kg^−1^ of Hg being present in soils from China, with a Hg concentration of 3.99 mg kg^−1^.

Rothenberg et al. [12] studied the behavior of Hg accumulation in the polished rice grain of 50 rice varieties grown at three different sites in Guizhou, China, that were classified as being highly contaminated (49,000.0 ± 2100 ng g^−1^), moderately contaminated (11,000.0 ± 1900 ng g^−1^), and background (170 ± 11 ng g^−1^), finding the highest values of Hg in the grain from moderately contaminated sites, with significant differences compared to the other sites. They emphasized that the values were 6.6 times higher than those of highly contaminated sites and 42 times higher than those of the background sites. This finding allowed the authors to conclude that the Hg concentration in the soil does not predict the Hg levels in the grain.

The root–grain translocation factor was less than one unit for all of the treatments (Figure 2d), decreasing as the mercury concentration in the soil increased. The highest translocation factor occurred for the FA2000 variety at the low Hg soil concentration, with 0.129, or approximately ten orders of magnitude higher than the rest of the treatments. These results reaffirm what was obtained for Hg accumulation in the grain, where it was found that there is a limited capacity to accumulate the metal in this organ for the three varieties of *O. sativa* L. studied here, even under scenarios in which the Hg concentration in the soil is ten orders of magnitude (1500 μg kg^−1^) higher than the values reported in the agricultural soils of the La Mojana region (130 μg kg^−1^).

Feng et al. [56] maintain that even when large quantities of Hg accumulate in the roots of rice plants, transport to the aerial organs is limited to the presence of the phytochelatins that form complexes with the metal as well as to the presence of other biomolecules with high potential to bind Hg. Other authors such as Meng et al. [57] argue that the inorganic Hg that reaches the grain can enter atmospherically, which has recently been confirmed by Strickman and Mitchell [58]. In an experiment involving inorganic mercury and methylmercury tracers, where it was observed that the limited translocation of Hg to the grain can be completely regulated by methylmercury levels in the soil, this chemical species of mercury was absorbed from the soil and bioaccumulate in the root and was finally translocated to the grain of *O. sativa* L. [59]. The previous statement is not intended to suggest that all of the Hg detected in the grains of this experiment is of an organic nature, as high temperatures in the greenhouse could promote increases in the levels of atmospheric mercury and the subsequent absorption of the metal by the plant.

### 3.6. Health Risk Due to Hg Rice Grain

The HQ values used to assess the potential health risk of rice intake are shown in Table 3. These values show that the intake of rice grown in Hg-contaminated soils for the different treatments evaluated here does not represent a health risk, with ratios lower than 0.36 in all cases and EWI values that are much lower than the provisional tolerable weekly intake (PTWI) of Hg (4 μg Hg/kg body weight/week) established by the JECFA [33]. The highest HQ values correspond to the FA473 variety grown in soils with a Hg concentration of 1500 μg kg^−1^, and the lowest values were found in the FAM variety grown in the soil with the lowest soil Hg concentration. The above findings suggest that the FAM variety can be cultivated in the soils of La Mojana region, reaching maximum HQ values similar to those found in FA2000 at high soil Hg levels but with minimum values at the ambient concentrations of the study soil.

## 4. Conclusions

In this experiment, three varieties of *Oryza sativa* L. were established, developed, and harvested in mercury-contaminated soils, which proved to be tolerant to the levels studied without visible phytotoxic damage. However, the FA2000 variety showed the lowest weight per 1000-grains under all of the evaluated Hg concentrations. The commercial varieties present differences in the mercury accumulation in their tissues, with the highest concentration being present in the roots. This accumulation led to an increase in grain Hg concentration, with the exception of the FA2000 variety, where the soil Hg concentration had no significant effect on grain Hg accumulation. All three varieties showed that Hg translocation from root to grain is lower at higher soil concentrations. The FA473 and FAM varieties showed the lowest risk in terms of rice intake at natural soil concentrations but increased Hg concentrations in the soil may represent a potential health risk to consumers of this grain. Therefore, under the current conditions found in La Mojana region, the cultivation of the FAM variety is suggested due to its yield component and lowest EWI values that are related to health risk.

## Figures and Tables

**Table 1:** Factorial ANOVA for biomass and weight per 1000 grains of commercial varieties when exposed to soils containing different Hg levels.

Source of Variation	Df	SS	F	*p*-Value
**Biomass**				
Variety	2	11,944.7	3.893	0.056
Concentration	2	14,434.4	4.704	0.036
Variety * Concentration	4	38,179.2	6.221	0.009
Error	10	15,341.7		
Total	18	79,900		
**Weight per 1000 grains**				
Variety	2	12.999	410.7	<0.001
Concentration	2	16.713	528.1	<0.001
Variety * Concentration	4	3.096	48.9	<0.001
Error	10	0.285		
Total	18	32.808		

* Df: degrees of freedom; SS: sum of squares; F: F-test; *p*-Value: probability.

**Table 2:** Factorial ANOVA for the Hg concentration in the roots, grains, and husks of the commercial varieties of *Oryza sativa* L. grown in soils containing different Hg levels (α = 0.05).

Source of Variation	Df	SS	F	*p*-Value
**Root**				
Variety	2	328,298	10.099	0.003
Concentration	2	4,316,391	132.787	<0.001
Variety * Concentration	4	848,646	13.054	<0.001
Error	11	178,784		
Total	19	5,698,977		
**Grain**				
Variety	2	301.318	12.824	0.001
Concentration	2	11.490	0.489	0.625
Variety * Concentration	4	155.978	3.319	0.047
Error	12	140.980		
Total	20	701.723		
**Husk**				
Variety	2	732.780	3.491	0.067
Concentration	2	1621.710	7.726	0.008
Variety * Concentration	4	1602.191	3.817	0.035
Error	11	1154.435		
Total	19	5294.442		

* Df: Degrees of freedom; SS: Sum of squares; F: F-test; *p*-Value: probability.

**Table 3:** HQ values for the different study treatments.

Variety	Hg Concentration in Soil (mg kg^−1^)	Mean HQ
FA2000	0.13	0.227 a
	0.8	0.218 a
	1.5	0.228 a
Total FA2000		0.224
FA473	0.13	0.116 b
	0.8	0.232 a
	1.5	0.350 c
Total FA473		0.232
FAM	0.13	0.099 b
	0.8	0.222 a
	1.5	0.241 a
Total FAM		0.183

Note: Different lowercase letters (a, b, c) indicate statistically significant differences (α = 0.05).

## Data Availability

Raw data that support the findings of this study available on request.
